# Genome-wide identification and integrated analysis of *TCP* genes controlling ginsenoside biosynthesis in *Panax ginseng*

**DOI:** 10.1186/s12870-024-04729-x

**Published:** 2024-01-13

**Authors:** Chang Liu, Tingting Lv, Yanhua Shen, Tao Liu, Mingming Liu, Jian Hu, Sizhang Liu, Yang Jiang, Meiping Zhang, Mingzhu Zhao, Kangyu Wang, Yi Wang

**Affiliations:** 1 College of Life Science, Jilin Agricultural University, Changchun, Jilin 130118 China; 2 Jilin Engineering Research Center Ginseng Genetic Resources Development and Utilization, Changchun, Jilin 130118 China

**Keywords:** *Panax ginseng*, TCP transcription factor, Ginsenoside biosynthesis, Expression pattern analysis, Methyl jasmonate treatment

## Abstract

*Panax ginseng* is an important medicinal plant, and ginsenosides are the main bioactive molecules of ginseng. The TCP (TBI, CYC, PCF) family is a group of transcription factors (TFs) that play an important role in plant growth and development, hormone signalling and synthesis of secondary metabolites. In our study, 78 *PgTCP* transcripts were identified from the established ginseng transcriptome database. A phylogenetic tree analysis showed that the 67 *PgTCP* transcripts with complete open reading frames were classified into three subfamilies, including CIN, PCF, and CYC/TB1. Protein structure analysis showed that *PgTCP* genes had bHLH structures. Chromosomal localization analysis showed that 63 *PgTCP* genes were localized on 17 of the 24 chromosomes of the Chinese ginseng genome. Expression pattern analysis showed that *PgTCP* genes differed among different lineages and were spatiotemporally specific. Coexpression network analysis indicated that *PgTCP* genes were coexpressed and involved in plant activities or metabolic regulation in ginseng. The expression levels of *PgTCP* genes from class I (PCF) were significantly downregulated, while the expression levels of *PgTCP* genes from class II (CIN and CYC/TB1) were upregulated, suggesting that *TCP* genes may be involved in the regulation of secondary metabolism in ginseng. As the *PgTCP26-02* gene was found to be related to ginsenoside synthesis, its predicted protein structure and expression pattern were further analysed. Our results provide new insights into the origin, differentiation, evolution and function of the *PgTCP* gene family in ginseng, as well as the regulation of plant secondary metabolism.

**Supplementary Information:**

The online version contains supplementary material available at 10.1186/s12870-024-04729-x.

## Introduction

Ginseng (*Panax ginseng* C. A. Meyer) is an important medicinal plant that has been widely studied in recent years [1, 2]. Ginsenosides are secondary metabolites and the main bioactive compounds that have medicinal value in ginseng [3]. Elicitors act as specific signals that induce the expression of target genes in cells, thereby regulating the synthesis of secondary metabolites in plant cells [4]. Methyl jasmonate (MeJA) is a volatile organic compound involved in plant defence and many different developmental processes, such as root growth, seed germination, fruit ripening, flowering and plant ageing [5]. It acts as an interplant signalling molecule that activates defence genes encoding proteins and secondary compounds (such as anthocyanins and alkaloids) [6]. The addition of exogenous MeJA has also been shown to increase the production of terpenoids in ginseng [7]. Due to its good and stable induction effect, MeJA has become one of the most commonly used inducers in the study of the ginsenoside synthesis pathway. In our study, MeJA was selected as an additional inducer to regulate ginsenoside biosynthesis.

The TEOSINTE BRANCHED1, CYCLOIDEA, and PCF (TCP) transcription factor gene family, containing plant-specific transcription factors [8], is widely involved in regulating plant seed germination [9], axillary meristem development [10–12], flower organ development [13], leaf morphogenesis [14], hormone signalling [15], and the synthesis of secondary metabolites [16–18]. TCP was first discovered in the 20th century in three species: teosinte Branched1 (TB1) from maize (*Zea Mays*) [19], CycloidEA (Cyc) from goldenseal [20], and proliferative cytokine (PCF) from rice (*Oryza sativa*) [21]. These gene family members all have a conserved TCP domain and a basic helix-loop-helix (bHLH) structure, which is mainly associated with DNA binding, protein interactions, and protein nuclear localization [22]. Based on the characteristics of the conserved structural domains of TCP proteins, they can be further divided into class I (also known as PCF) and class II (including the CIN and CyC/Tb1 subfamilies) [23].

Some *TCP* gene family members can participate in the regulation of secondary metabolite synthesis in plants [24]. Current studies on *TCP* and secondary metabolism mainly focus on transcriptome analysis [25]. Li et al. studied miRJAW-resistant *AtmTCP3* transgenic plants and *AtTCP3SRDX* mutant plants with an inactivated *AtTCP3* gene [16] and found that the seedlings and seeds of *AtmTCP3* plants had excessive accumulation of flavonols, anthocyanins and proanthocyanidins, while the levels of proanthocyanidins in *TCP3SRDX* plants decreased slightly. In addition, the R2R3-MYB protein activated late flavonoid biosynthesis genes by forming the terpolymer R2R3-MYB/bHLH (TCP3)/WD40 (MBW) complex, indicating that *TCP* can promote flavonoid biosynthesis. The *TCP* gene family has been extensively studied in other species, such as *Arabidopsis* [22], rice [26], cotton [15], chrysanthemum [27], soybean [28], tomato [29], ginkgo [24], bamboo shoots [30], grapes [31], Dendrobium [32], orchids [33], and ginseng [34]. However, this gene family has not been screened for candidate genes involved in ginsenoside biosynthesis.

In this study, 78 *PgTCP* transcripts under 28 *PgTCP* gene IDs were identified from the transcriptome database of ginseng and classified according to their domain information (CIN, PCF, and CYC/TB1). Then, we analysed the evolutionary relationships and conserved motifs of the *PgTCP* gene family, annotated them with GO function, and analysed expression patterns and coexpression networks based on PgTCP gene expression data. Then, the response of *PgTCP* gene family members to different treatment times of methyl jasmonate (MeJA) was explored. Finally, we identified a gene that was significantly related to ginsenoside biosynthesis.

## Materials and methods

### Identification of the *TCP* gene family in ginseng

To ensure the completeness and accuracy of the *TCP* gene family, we used different methods to identify the *TCP* gene family. First, the Jilin Ginseng Transcriptome Database [35] was used as a query sequence for searching ginseng *TCP* transcripts with an e value of 1 × 10^–6^. Second, the hidden Markov model (HMM) of *TCP* genes was downloaded from Pfam (Pfam ID: PF03634), and potential *TCP* genes were identified from the Jilin ginseng transcriptome database using HMMER (http://HMMER.janelia.org) with an *E-value* of 1.0E-06. Finally, TCP amino acid sequences were downloaded from the Plant Transcription Factor Database (http://planttfdb.gao-lab.org/family.php?fam=TCP) as BLAST query sequences and use to search the Jilin ginseng transcriptome database. The results of the three methods were then combined, and duplicates were removed. The results were submitted to iTAK (http://itak.feilab.net/cgi-bin/itak/index.cgi) to exclude some spurious sequences. Finally, NCBI CD-Search (http://www.ncbi.nlm.nih.gov/Stru-cture/cdd/wrpsb.cgi) and SMART online tool (https://smart.embl.de/) were used to confirm the presence of the candidate gene transcripts in TCP conserved structural domains, and transcripts containing TCP conserved structural domains were selected and defined as *PgTCP* gene transcripts. Arabic numbers were added to *PgTCP*, e.g., *PgTCP01*, to indicate different gene serial numbers. A suffix (e.g., -01) was used to indicate different transcripts. The online software ExPASy-Prot Param tool (https://web.ExPASy.org/protparam/) was used to predict the basic physicochemical properties of the PgTCP proteins, including theoretical molecular weights (kDa) and isoelectric points (PI).

### Phylogenetic analysis, conserved domain and motif analysis of *PgTCP* transcripts

To classify the *PgTCP* transcripts, we compared the 67 *PgTCP* transcripts with complete TCP structural domains with three other species. Dicots, monocots, and model plants were selected as outgroup species, and nine *TCP* genes of three species, *Oryza sativa* (*Os*), *Arabidopsis thaliana* (*At*), and *Solanum lycopersicum* (*Sl*), which were downloaded from NCBI for phylogenetic analysis with *PgTCP* genes. Phylogenetic trees were constructed using the maximum likelihood (ML) method in MEGA-X [36], and bootstrap replicates were set to 2000. The final evolutionary trees were edited using the Evolview version 3.0 online website (https://www.evolgenius.info/evolview/#login) [37]. We performed conserved motif analysis using MEME (http://meme.nbcr.net/meme/) [38]. The maximum and minimum conserved motif lengths were 10 and 50 amino acids, respectively.

### Protein structure analysis of the *PgTCP* gene family in ginseng

SOPMA (https://npsa-prabi.ibcp.fr/cgi-bin/npsa_automat.pl?page=/NPSA/npsa_sopma.html) [39] and SWISS-MODEL (https://swissmodel.ExPASy.org/), two online software programs, were used to analyse the protein secondary and tertiary structures of the amino acid sequences of PgTCP genes in ginseng, respectively.

### Chromosome localization and covariance analysis

We used BLASTN to compare the above *PgTCP* genes with ginseng genomes [40]. Identity ≥ 95%, coverage length ≥ 300 bp and *E-value* ≤ *1.0E-100* were used as criteria. The position of *PgTCP* transcripts on chromosomes was visualized using the MG2C online tool (http://mg2c.iask.in/mg2c_v2.1/index.html). The *PgTCP* gene family and ginseng genome were subjected to covariance analysis, and the repeated genes of the *PgTCP* gene in the ginseng genome were analysed by the R package circlize34 to determine the pan transcription and core transcription of the *PgTCP* gene family in the ginseng genome.

### GO (Gene Ontology) annotation, functional classification, and analysis

We annotated and classified the identified *PgTCP* transcripts in GO using Blast2GO version 6.0.3 [41] and used the EVeen online tool (http://www.ehbio.com/test/venn/#/) for visualization and analysis. The results of annotation and GO classification were used to assess the functional differentiation of *PgTCP* genes. The chi-square test at level 2 was used to determine the number of *PgTCP* transcripts involved in specific functions and the number of transcripts involved in multiple functions, and R language was used to show the genetic ontological annotation of the ginseng *TCP* gene family.

### Expression pattern analysis of the *PgTCP* gene family

To analyse the expression patterns of *PgTCP* transcripts, we determined the expression of *PgTCP* in 14 different tissues, 4 different aged stages (5, 12, 18, and 25 years) and 42 farm cultivars of 4-year-old ginseng roots. The expression heatmap and gene visualization heatmap were constructed using the R language package for *PgTCP* gene expression in 14 different tissues, 4 different aged stages of ginseng roots to show the spatiotemporal characteristics of *PgTCP*, and 4-year-old ginseng roots of 42 farm cultivars to reveal the characteristics among different genotypes.

To further investigate the interaction characteristics between the expression of *PgTCP* genes in 42 farm cultivars, Spearman correlation coefficients were calculated using the R programming language and software (http://www.rproject.org/). Gene coexpression networks were constructed using BioLayout Express ^3D^ version 3.2 software [42].

### Plant materials and methyl jasmonate treatment

Ginseng hairy root was obtained from Jilin Engineering Research Center Ginseng Genetic Resources Development and Utilization. A 0.2 g sample of ginseng hairy root was inoculated into a 250 mL flask containing 150 mL 1/2 MS liquid medium, placed in a shaker at 22 °C and shaken at 110 rpm. On Day 23, MeJA was added to the culture vial. Each trial group included three replicates and one control. The dosage of MeJA was 200 µM. At each time point of 6, 12, 24, 48, 72, 96 and 120 h, three biological replicates and one blank control were collected, and the blank control was not treated with MeJA. Ginseng hairy root samples were quickly frozen in liquid nitrogen for subsequent experiments [43].

### RNA extraction and qRT‒PCR validation

Total RNA of ginseng was extracted by the TRIzol method and reverse transcribed into cDNA. According to the basic principle of primer design, the most suitable primer was designed to perform qRT‒PCR on the cDNA of ginseng hairy root. *GAPDH* (glyceraldehyde-3-phosphate dehydrogenase) was selected as the internal reference gene based on the pretest screening, and fluorescence quantitative PCR was performed using the SYBR Premix Ex Taq™ II (Tli RNaseH Plus) kit. The reaction system was as follows: 5.0 µL SYBR PreMix Ex Taq II, 0.4 µL Sense Primer, 0.4 µL Anti primer, 0.5 µL cDNA and 3.7 µL ribonuclease-free water. The reaction conditions were as follows: predenaturation at 95 ℃ for 30 s; reaction at 95 ℃, 5 s; 60 ℃, 34 s, 40 cycles; solution curve 95 ℃, 15 s; 60 ℃, 1 min; 95 ℃, 15 s. Technical experiments were repeated three times for each group of samples, and the final results were calculated by the 2^−ΔΔCT^ method [44].

### Identification of candidate genes related to ginsenoside biosynthesis

The expression data of *PgTCP* and the expression data of each mono saponin and total saponins in 42 farm cultivars of ginseng in Jilin Province were sorted. Pearson correlation coefficient analysis was conducted using SPSS software to calculate the correlation between *PgTCP* and ginsenoside synthesis, and the closely related genes were screened out. The expression data of *PgTCP* genes significantly related to ginsenoside synthesis and the gene expression data of 16 key enzymes involved in the ginsenoside synthesis pathway were sorted. Pearson correlation coefficient analysis was conducted using SPSS software to calculate the correlation between *PgTCP* and key enzyme-encoding gene expression. Closely related genes were screened out. Spearman’s correlation coefficients were calculated using the R programming language and software (http://www.rproject.org/). BioLayout Express^3D^ version 3.2 software was used to construct the coexpression network of the *PgTCP* gene and ginsenoside synthesis key enzyme-encoding genes and visualize the gene interaction network.

### Characteristic analysis of the *PgTCP26-02* gene involved in ginsenoside biosynthesis

Protein secondary structure analysis of the amino acid sequence of *PgTCP26-02* was performed by SOPMA (https://npsa-prabi.ibcp.fr/cgi-bin/npsa_automat.pl?page=npsa_sopma.html) online software. Using SWISS-MODEL (https://www.swissmodel.ExPASy.org/), online software was used to analyse the tertiary structure of the protein based on the amino acid sequence of PgTCP26-02. Phylogenetic trees were constructed with the neighbour-joining (NJ) method in MEGA-X using the protein sequences of PgTCP26-02 and those of other species, and the bootstrap replicates were set to 2000. The protein sequences of PgTCP26-02 and the other three species were aligned using DNAMAN software.

In addition, to further analyse the expression pattern of the *PgTCP26-02* gene, TBtools version 2.012 [45] was used to generate a heatmap of *PgTCP26-02* gene expression in ginseng roots of 4 different ages (5, 12, 18, 25 years old), 14 different tissues of 4-year-old ginseng and 42 farm cultivars of 4-year-old ginseng to observe the expression of the *PgTCP26-02* gene in ginseng more clearly and intuitively.

## Results

### Genome-wide identification of the *TCP* gene family in ginseng

A total of 574 transcript sequences containing TCP structural domains were identified from the Jilin Ginseng Transcriptome Database using different methods after removing repetitive sequences. After iTAK filtering for spurious sequences, conserved structural domain analysis by NCBI CD-Search and SMART online software, 496 conserved structural domains among the 574 transcripts were incomplete or had no open reading frame (ORF), and the remaining 78 transcripts contained TCP structural domains in their ORFs. Therefore, these 78 transcripts were used for subsequent analysis. The 78 Jilin Ginseng *PgTCP* transcripts were classified as 28 *PgTCP* genes, named *PgTCP01*-*PgTCP28*. Different transcripts of the same gene were distinguished by numerical suffixes (e.g., − 01). These transcripts ranged in length from 229 to 3140 and in amino acid numbers from 29 to 435 for the complete open reading framework (ORF) (Table S1).

### Structural characteristics and phylogeny of *PgTCP* genes

**Fig. 1:**
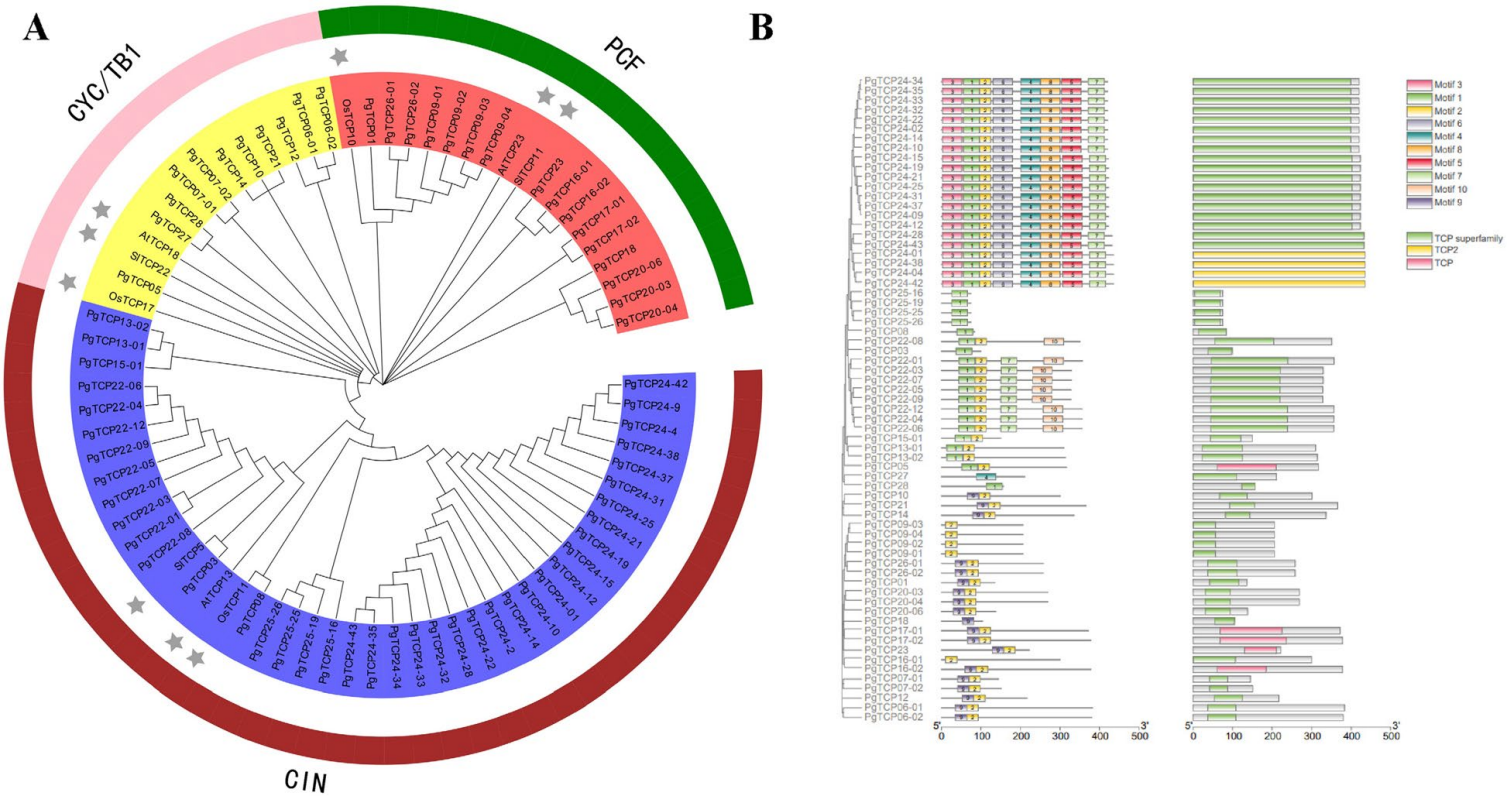
Phylogenetic analysis and conserved motifs analysis. **A** Phylogenetic analysis of the *PgTCP* genes, the stars represents three exogenous species. **B** Conserved motif analysis of *PgTCP*, different colors represent different conserved motifs

We constructed a phylogenetic tree by selecting 67 *PgTCP* transcripts with intact TCP structural domains and 9 transcripts of other species (Table S2). The results are shown in Fig. 1A. *PgTCP* transcripts were divided into class I (PCF) and class II, which were further divided into the CYC/TB1 class and CIN class.

To understand the sequence characteristics of PgTCP proteins, the online tool MEME was used to analyse its conserved domain. The results showed that motif number ranged from 1 to 8 in subfamily members of different *PgTCP* genes (Fig. 1B). The conserved structural domains of TCP were classified into three types, namely, the TCP superfamily, TCP2 and TCP, which were expressed in all 67 *PgTCP* family members. These results suggest that *PgTCP* genes are functionally similar.

### Protein structure analysis of *PgTCP* transcription factors in ginseng

**Fig. 2:**
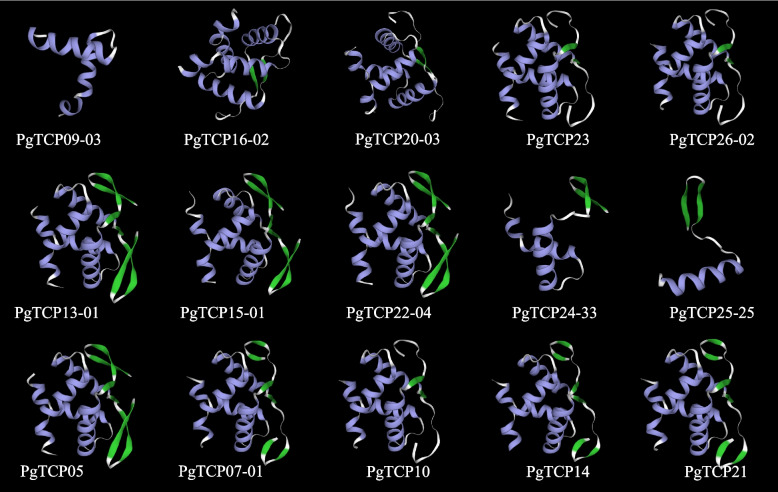
Tertiary structure analysis of ginseng *TCP* family members. The secondary structure elements include alpha helix, beta turn, and random coil. Purple represents the Alpha helix, green represents the Beta turn, gray represents the Random coil

Five genes from each of the three isoforms of the *PgTCP* gene family were selected and analysed for their secondary structures as well as tertiary structures. The predicted secondary structures of the 15 proteins showed that the TCP proteins consisted of four parts: α-helix, extended chain, random coiled coil, and β-turn. The ginseng TCP protein had the highest proportion of irregularly coiled structures, followed by α-helix structures (Table 1). As shown in Fig. 2, the tertiary structure of ginseng TCP-encoded proteins was as follows: ginseng TCP protein contains alpha helix, beta turn, and random coil structures. Different proteins have different structures, indicating that their functions are also different, further indicating the functional diversity of TCP gene family members.

**Table 1:** Secondary structure of PgTCP protein

Protein ID	Alpha helix		Extended strand		Beta turn		Random coil	
	**Number of amino acids**	**%**	**Number of amino acids**	**%**	**Number of amino acids**	**%**	**Number of amino acids**	**%**
PgTCP09-03	50	24.27	24	11.65	6	2.91	126	61.17
PgTCP16-02	92	24.34	42	11.00	18	4.76	226	59.79
PgTCP20-03	77	28.62	21	7.81	14	5.20	157	58.36
PgTCP23	32	14.41	37	16.67	10	4.50	143	64.41
PgTCP26-02	56	21.71	28	10.85	12	4.65	162	62.79
PgTCP13-01	41	13.23	36	11.61	8	2.58	225	72.58
PgTCP15-01	41	27.33	26	17.33	7	4.67	76	50.67
PgTCP22-04	47	13.20	44	12.36	10	2.81	255	71.63
PgTCP24-33	77	18.33	49	11.67	6	1.43	288	68.57
PgTCP25-25	25	33.33	19	25.33	6	8.00	25.00	33.33
PgTCP05	137	42,27	26	8.20	5	1.58	152	47.95
PgTCP07-01	33	22.76	9	6.21	8	5.52	95	65.52
PgTCP10	45	14.95	40	13.29	10	3.32	206	68.44
PgTCP14	41	12.20	41	12.20	12	3.57	242	72.02
PgTCP21	63	17.21	52	14.21	19	5.19	232	63.39

### Chromosome distribution and covariance analysis

**Fig. 3:**
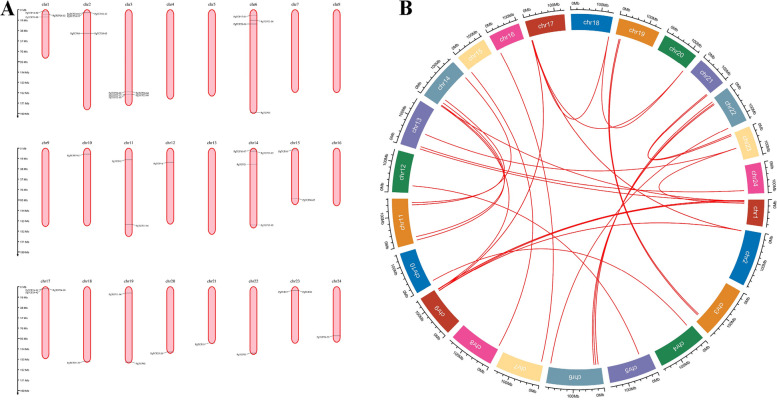
*PgTCP* chromosome distribution and covariance analysis. **A** Distribution of ginseng *TCP* family members on ginseng chromosomes. **B** Covariance analysis of ginseng *TCP* gene family members in ginseng chromosomes. The red line represents tandem duplication of the same gene on different chromosomes

Of the 78 *PgTCP* genes, 63 were localized on Chinese ginseng genome chromosomes after comparison with 24 ginseng chromosomes. Among the 63 *PgTCP* genes localized to the Chinese ginseng genome, no TCP members were identified on chromosomes 4, 5, 7, 8, 9, 13, and 16, as shown in Fig. 3A. In ginseng, the chromosomal distribution of *PgTCP* members was uneven. Chromosome 2 contained the most *PgTCP* members (13). Covariance analysis showed that members of the *TCP* gene family had undergone gene replication in ginseng (Fig. 3B).

### GO functional categorization and GO term enrichment of *PgTCP* genes

**Fig. 4:**
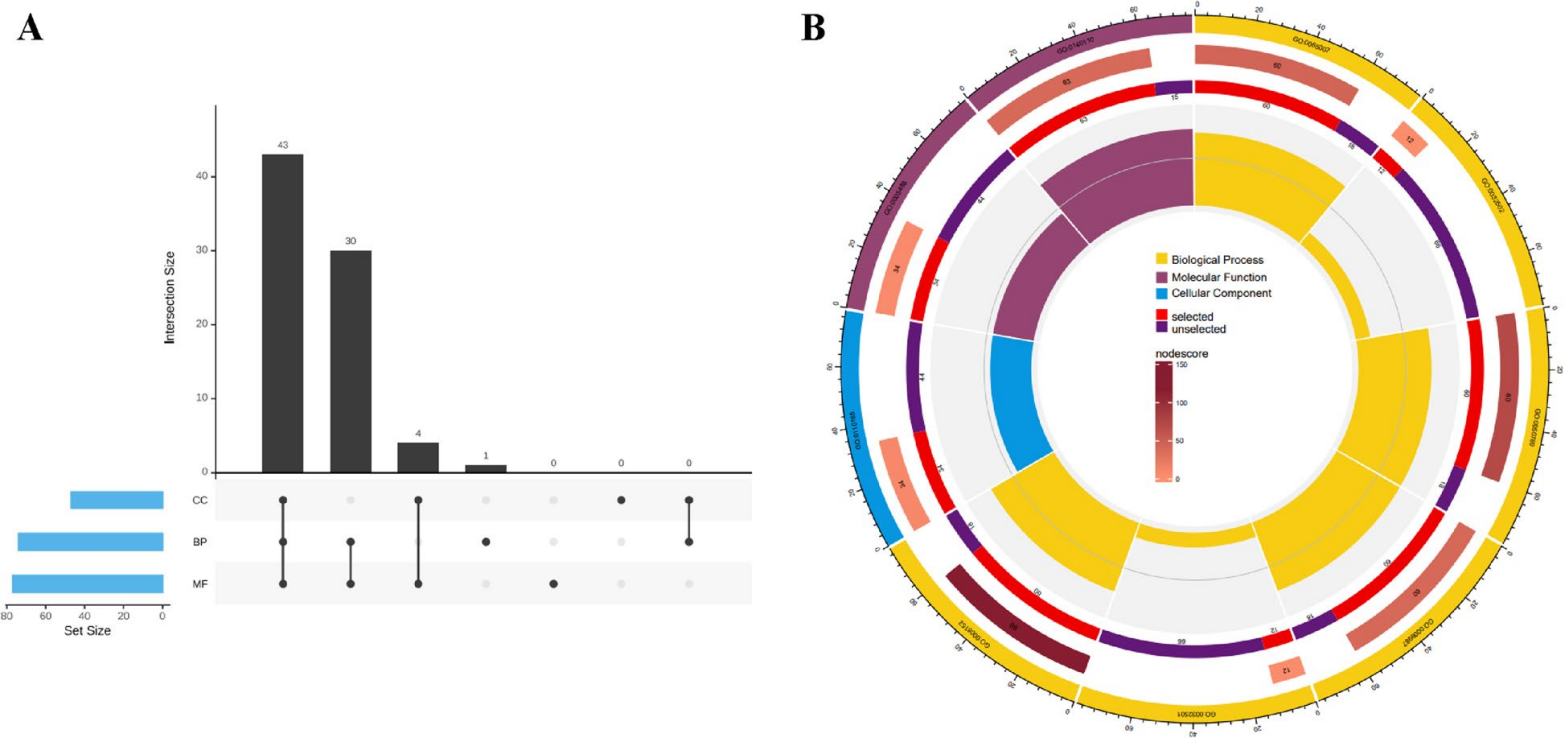
Functional categorization and GO term enrichment of the *PgTCP* gene transcripts. **A** Venn network of the *PgTCP* gene transcripts among the biological process (BP), molecular function (MF) and cellular component (CC) functional categories. **B** The *PgTCP* transcripts are classified into nine functional categories at Subcategories (Level 2), including one CC functional categories (Blue), two MF functional category (Purple), and six BP functional categories (Yellow)

In living organisms, genes usually have multiple functions. We performed GO annotation of 78 *PgTCP* transcripts (Table S3). We found that all 78 transcripts had at least one of three major functions: 74 were biological processes (BP), 47 were cellular components (CC), and 77 were molecular functions (MF). Only one function was labelled in 1 transcript, two major functions were labelled in 34 transcripts, and three major functions were labelled in 43 transcripts (Fig. 4A). At level 2, 6 sublevels, GO:0065007 (biological regulation), GO:0032502 (developmental process), GO:0050789 (regulation of biological process), GO:0009987 (cellular process), GO:0032501 (multicellular organismal process), and GO:0008152 (metabolic process), were enriched in BP, 1 sublevel, GO:0110165 (cellular anatomical entity), was enriched in CC, and 2 sublevels, GO:0005488 (binding) and GO:0140110 (transcription regulator activity), were enriched in MF (Fig. 4B).

### Expression characteristics and pattern of *PgTCP* genes

To further understand the regularity of *PgTCP* gene expression in ginseng, we retrieved the expression data of 78 *PgTCP* gene transcripts from 42 farm cultivars (S1—S42), 14 different tissues (fibre root, leg root, main root epiderm, main root cortex, rhizome, arm root, stem, leaf peduncle, leaflet pedicel, leaf blade, fruit peduncle, fruit pedicel, fruit flesh, and seed), and four different ages (5, 12, 18, and 25 years) of ginseng roots (Table S4) and plotted heatmaps.

**Fig. 5:**
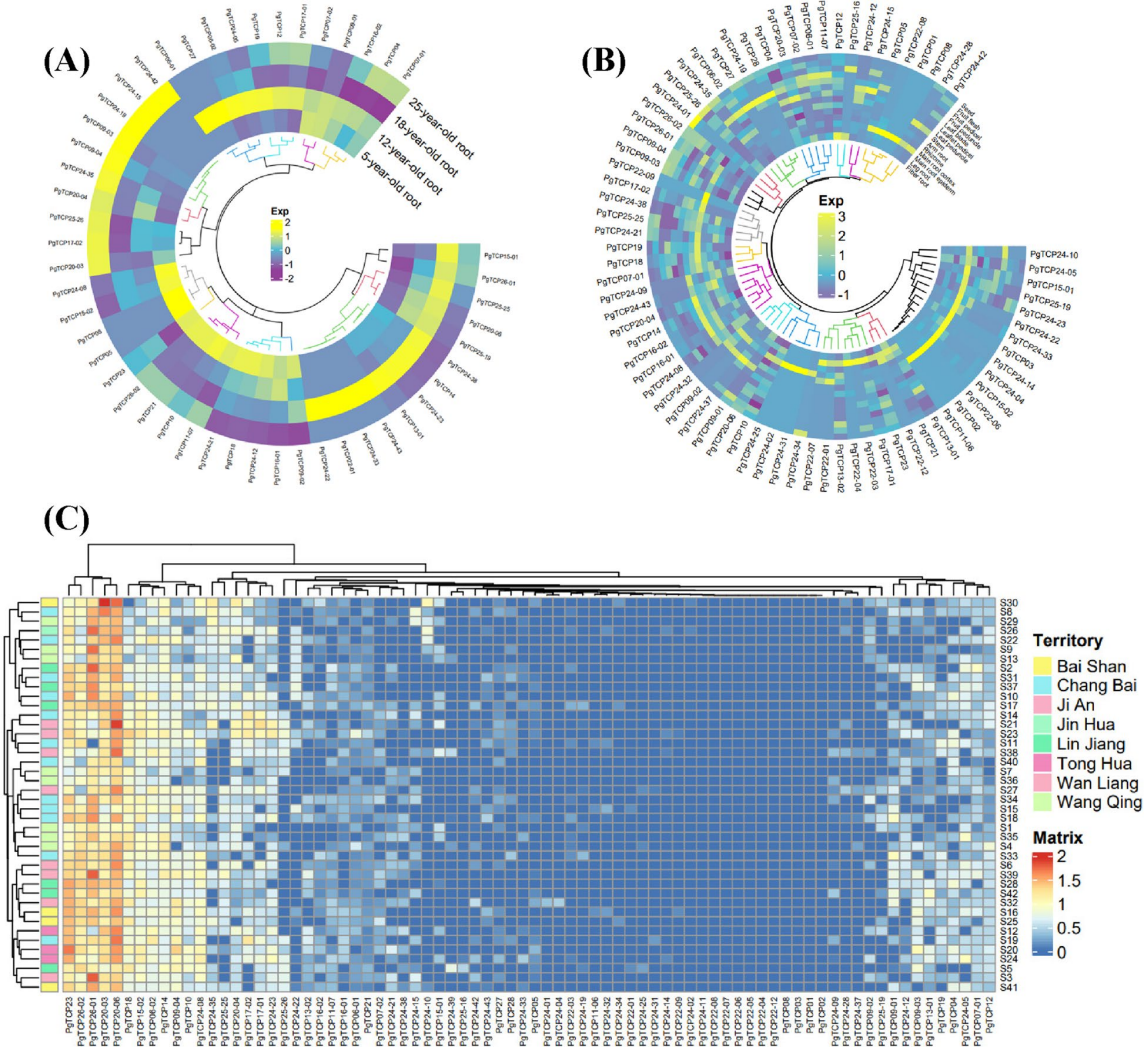
Heatmaps analysis spatiotemporal expression patterns of *PgTCP* transcripts in *Panax ginseng*. **A** The *PgTCP* genes expression in the 4 different aged (5, 12, 18, 25 years) of ginseng roots. **B** The *PgTCP* genes expression in the 14 different tissues of 4-year-old ginseng. **C** The *PgTCP* genes expression in the 42 farm cultivars of 4-year-old ginseng roots

The results showed that 29 transcripts (37%) were not expressed in the roots of ginseng at 4 different ages. Heatmaps of the remaining gene expression showed that 22 *PgTCP* transcripts were expressed in all 4 age groups (28%), and 49 *PgTCP* transcripts (62%) were expressed in at least one age group (Fig. 5A). *PgTCP20-06*, *PgTCP26-01*, *PgTCP18*, *PgTCP24-8*, *PgTCP14*, *PgTCP20-03*, *PgTCP17-02*, *PgTCP17-01* and *PgTCP20-04* were highly expressed.

Among 78 transcripts, 3 transcripts were not expressed in 14 different tissues of four-year-old ginseng, and heatmap results after the deletion of nonexpressed transcripts showed that 14 transcripts (18%) were expressed in all 14 tissues and 75 *PgTCP* transcripts were expressed in at least one tissue (96%) (Fig. 5B). *PgTCP20-03* and *PgTCP20-06* were expressed at higher levels.

The heatmap analysis of 42 farm cultivars of 4-year-old ginseng roots showed that 67 *PgTCP* transcripts were expressed in at least one cultivar (86%) (Fig. 5C). The *PgTCP23*, *PgTCP26-01*, *PgTCP26-02*, *PgTCP20-03*, and *PgTCP20-06* genes had high expression levels in 42 farm cultivars, and the expression levels of 11 transcripts in all cultivars were zero. This indicates that there are regional differences in the expression of *TCP* gene family members in ginseng.

### Coexpression network of *PgTCP* transcripts

**Fig. 6:**
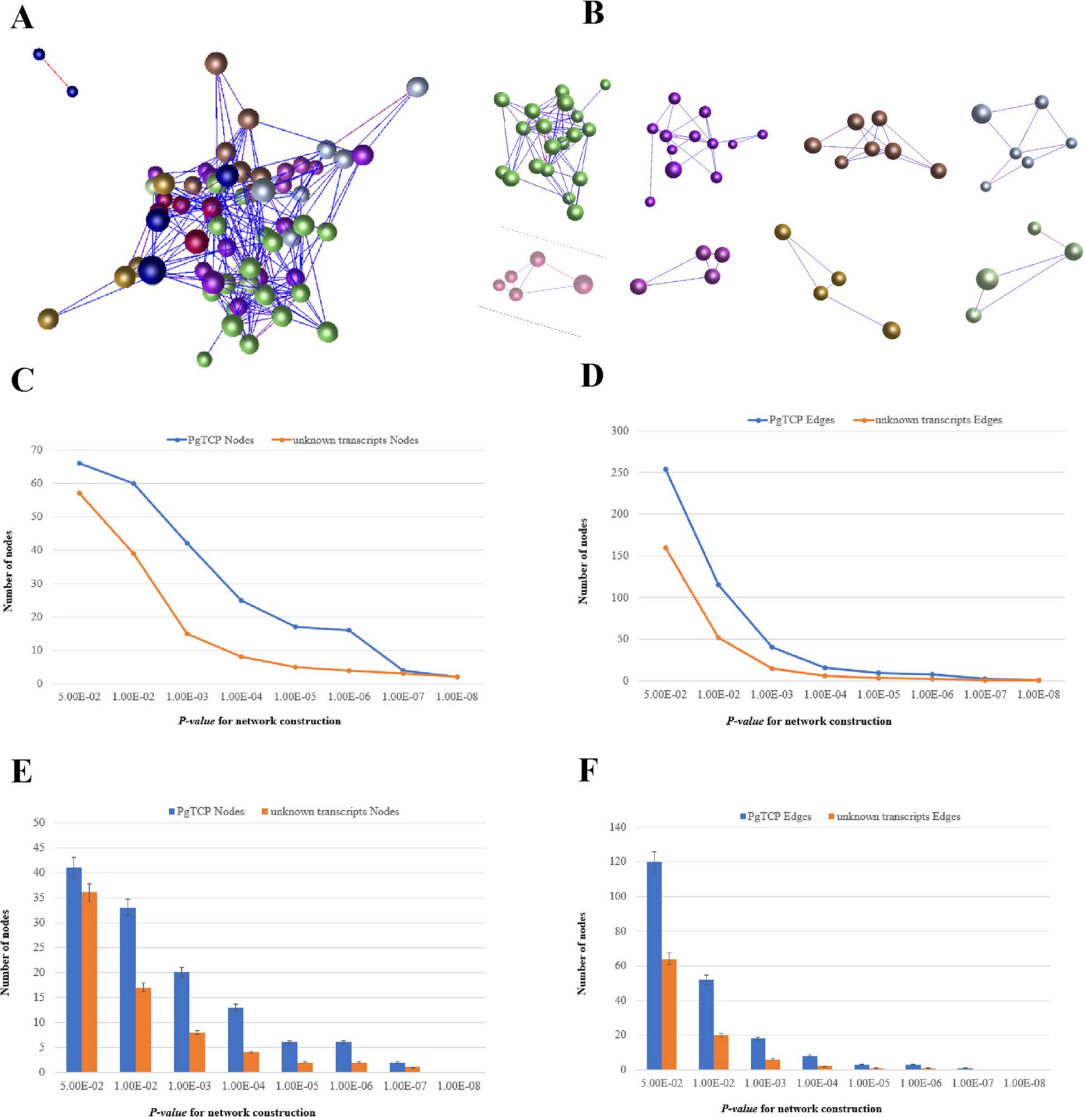
Network analysis of the *PgTCP* genes expressed in the 4-year-old roots of 42 farm cultivars. **A** The co-expression network constructed from the 78 *PgTCP* transcripts. The network was constructed at *P* ≤ 5.0E-01. **B** The three clusters constituting the network. Different clusters are indicated by different colors. **C** Tendency that these *PgTCP* form a network, with the randomly-selected ginseng unknown genes as controls: variation in number of nodes. **D** Tendency that these Pgtcp transcripts form a network, with the randomly-selected ginseng unknown genes as controls: variation in number of edges. **E** Statistical analysis of variation in number of nodes in the network. **F** Statistical analysis of variation in number of edges in the network. Different capital letters, significant at *P* ≤ 0.01. Error bar, standard deviation for 20 replications

To investigate whether there are correlations between different gene types in *PgTCP* transcripts, coexpression network analysis was performed for *PgTCP* transcript expression levels in 42 cultivars. Sixty-seven transcripts expressed in at least one of 42 cultivars were selected for coexpression network analysis. The coexpression network results showed that at *P* ≤ *5.0E-02*, the 67 transcripts formed a coexpression network with 254 edges and 66 nodes (Fig. 6A), and the network contained 8 clusters (Fig. 6B). We counted the nodes and edges of this network at increasingly stringent *P values*, and at *P* ≤ *1.0E-08*, the *PgTCP* transcripts formed 2 nodes and 1 edge. To reflect the tightness of this network, we randomly selected another 78 transcripts from the ginseng transcriptome database as negative controls, and we removed those that were not expressed in the 42 farm cultivars and selected the remaining ones for coexpression network analysis (Fig. 6C-D). After three replicates, the mean was calculated, and at *P* ≤ *5.0E-02*, the *PgTCP* transcript formed a regulatory network consisting of 57 nodes and 159 edges, and at *P* ≤ *1.0E-08*, the *PgTCP* transcript formed 2 nodes and 1 edge. Thus, *PgTCP* transcripts are more likely to form a coexpression network than randomly selected transcripts.

To further demonstrate the correlation between each pair of *PgTCP* transcripts, we constructed the network using a random sampling of two-thirds (52) of the total *PgTCP* transcripts and introduced a negative control as described above (Fig. 6E-F). When *P* ≤ 5.0E-02, 52 *PgTCP* transcripts formed a network of 120 edges and 41 nodes after removing unexpressed transcripts from 42 varieties, and at *P* ≤ *1.0E-07*, *PgTCP* transcripts formed 2 nodes and 1 edge, and the number of edges for the unknown transcripts was 0. These results suggest that there are significant coexpression interactions between *PgTCP* transcripts and that *PgTCP* transcripts form coexpression networks more easily than transcripts selected at random.

### Expression analysis of *PgTCP* genes under MeJA treatment in *Panax ginseng*

**Fig. 7:**
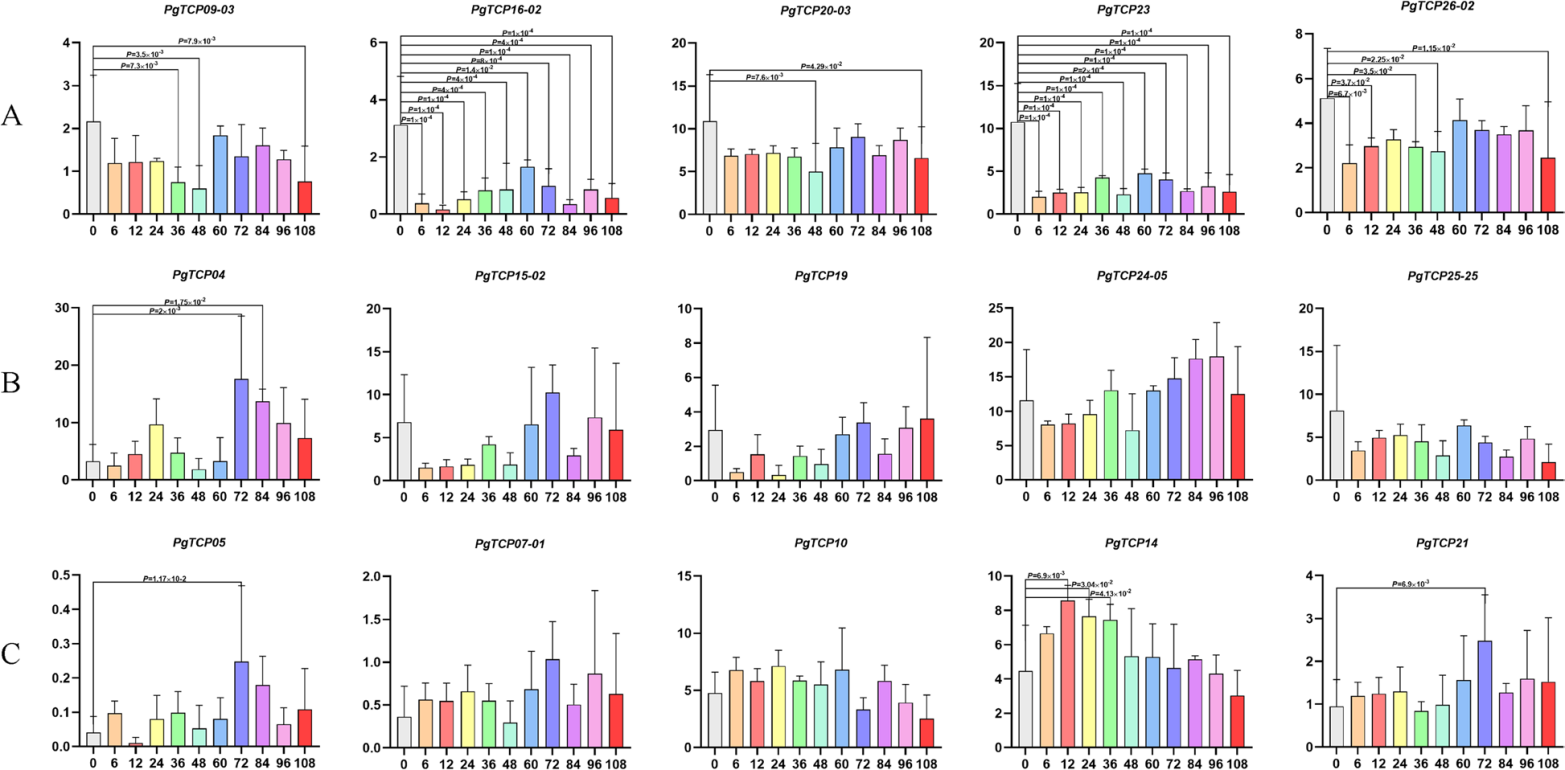
Relative expression of *PgTCP* in methyl jasmonate-treated ginseng. **A** Relative expression of PCF isoforms of *PgTCP* in methyl jasmonate-treated ginseng. **B** Relative expression of CIN isoforms of *PgTCP* in methyl jasmonate-treated ginseng. **C** Relative expression of CYC/TB1 isoforms of *PgTCP* in methyl jasmonate-treated ginseng. X shows the time (h) of methyl jasmonate-treated of ginseng hairy roots; Y represents the relative expression levels of genes in the hairy roots of ginseng

To determine the expression profile of the *PgTCP* gene under MeJA treatment, qRT‒PCR was performed on 15 transcripts randomly selected in 3.2 to explore the expression of the *PgTCP* gene under MeJA treatment at different times. As shown in Fig. 7, the expression levels of *PgTCP* genes in class I (PCF) were downregulated under MeJA treatment compared with the control, and the expression of most *PgTCP* genes showed an upwards and then downwards trend after MeJA treatment, reaching a peak at 60 h after induction. At 48 h after induction, the expression levels of the *PgTCP09-03*, *PgTCP16-02*, *PgTCP20-03*, *PgTCP23*, and *PgTCP26-02* genes were significantly downregulated compared with those of the control, and the relative expression levels of the *PgTCP16-02* and *PgTCP23* genes were significantly downregulated at all time points. In class II (CIN and CYC/TB1), almost all expression levels of *PgTCP* genes were upregulated compared with the control, and the expression of most *PgTCP* genes showed an upwards and downwards trend after MeJA treatment. Among the genes of the CIN subtype, the *PgTCP04*, *PgTCP15-02*, and *PgTCP19* genes peaked at 72 h after induction, and only the *PgTCP04* gene was significantly upregulated at 72 h. Among the CYC/TB1 subtypes, the relative expression levels of the *PgTCP05*, *PgTCP07-01*, and *PgTCP21* genes peaked at 72 h postinduction, and the *PgTCP05* and *PgTCP21* genes were significantly upregulated. The *PgTCP14* gene was significantly upregulated at 12 h postinduction and peaked at 12 h.

### Screening of *TCP* candidate genes involved in ginsenoside biosynthesis

Ginsenoside is the main active ingredient of ginseng, but its content in ginseng is very low, so it is very important to study the synthetic pathway of ginsenoside. SPSS software was used to calculate the correlation between ginsenoside content and *PgTCP* gene expression in 42 farm cultivars (Table S5). Twenty-nine *PgTCP* genes were significantly correlated with ginsenoside content, among which 19 *PgTCP* genes were significantly positively correlated with saponin content, and 10 *PgTCP* genes were significantly negatively correlated with saponin content.

Many key enzymes in ginsenoside synthesis have been cloned. The *PgTCP* gene may be correlated with key enzyme-encoding genes in the ginsenoside synthesis pathway. To explore the relationship between the *PgTCP* gene and key enzyme-encoding genes, SPSS software was used to calculate the correlation between the expression levels of key enzyme-encoding genes and the expression levels of 29 *PgTCP* genes significantly correlated with ginsenoside content (Table S6). A total of 19 genes were found to be significantly correlated with the expression of key enzyme-encoding genes. The expression levels of 16 *PgTCP* genes were positively correlated with the expression levels of key enzyme-encoding genes. The expression levels of three *PgTCP* genes were negatively correlated with the expression levels of key enzyme-encoding genes.

**Fig. 8:**
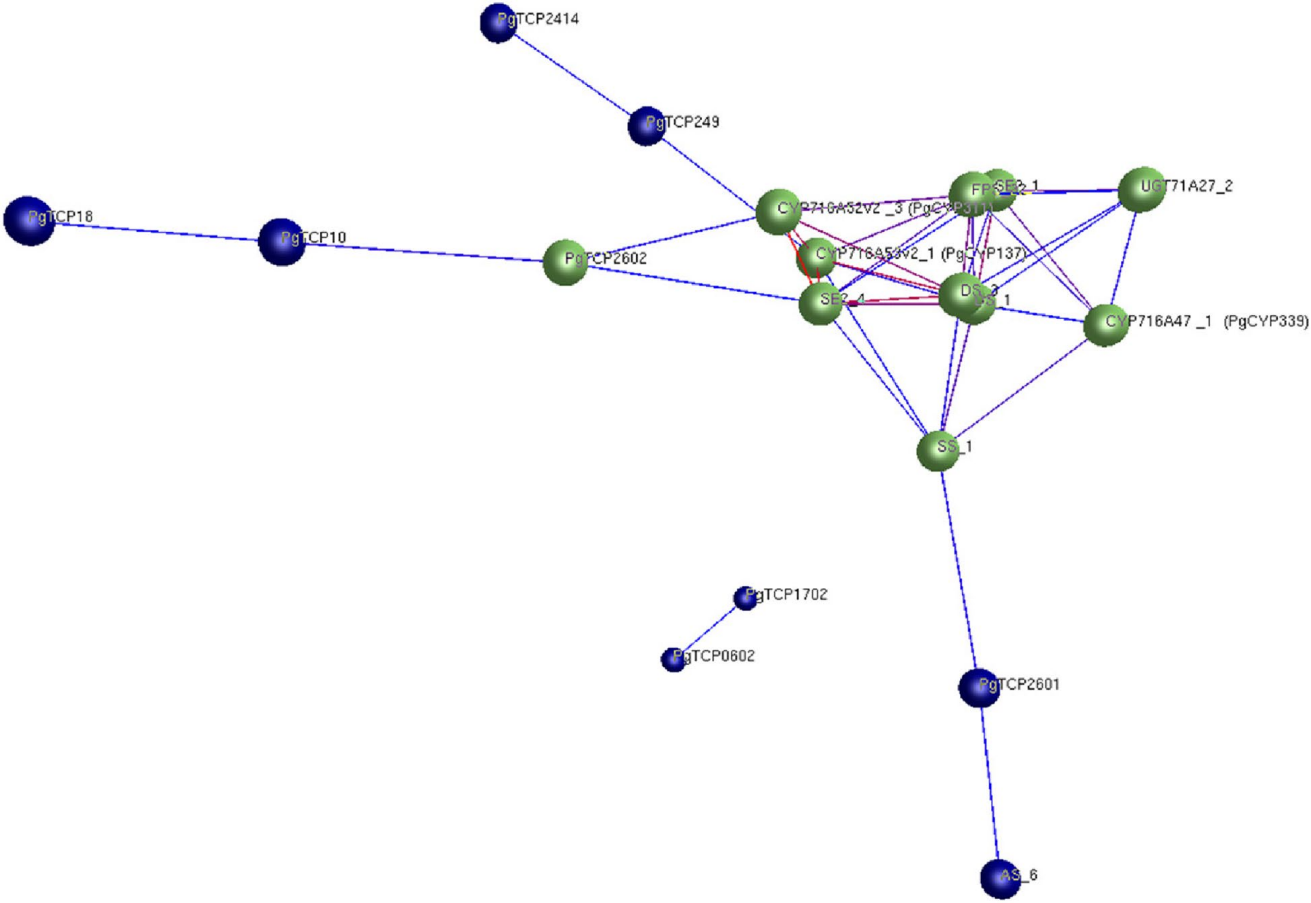
Interaction network between *PgTCP* genes and ginsenoside synthesis key enzyme genes

Since the key enzyme-encoding genes of ginseng participate and are important in the ginsenoside synthesis pathway, it is particularly important to study the correlation between key enzyme-encoding gene expression and ginsenoside content. After screening, 19 *PgTCP* genes were found to be significantly correlated with both key enzyme-encoding genes of ginseng and ginsenoside content. However, based on the network, the *PgTCP26-02* gene was associated with more key enzyme-encoding genes (Fig. 8), which was finally selected for subsequent functional verification.

### Analysis of the *PgTCP26-02* gene involved in ginsenoside biosynthesis

**Fig. 9:**
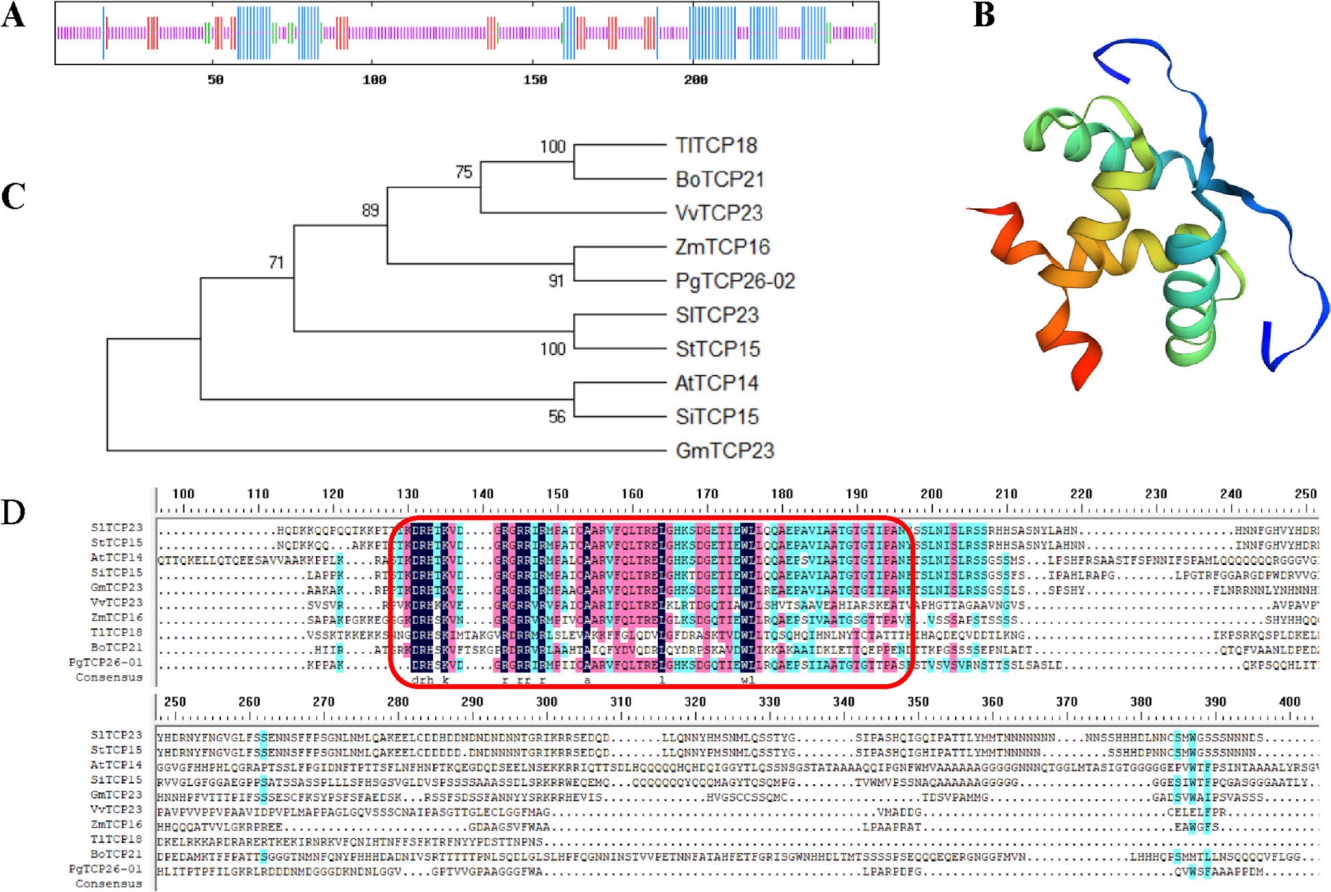
Characterization of the *PgTCP26-02* gene. **A** Secondary structure of PgTCP26-02 protein. The blue, green, purple and red lines represent alpha helix, beta turn, random coil and extended strand. **B** Tertiary structure of PgTCP26-02 protein. **C** Evolutionary relationships between PgTCP26-02 protein and TCP protein in other species. **D** Amino acid sequence alignment of PgTCP26-02 to protein sequences of other species. In the red box are the bHLH domains

The secondary structure of *PgTCP26-02* was α helix 56 (21.71%). β turn 12 (4.65%); random coil 162 (62.79%); extended strand 28 (10.85%) (Fig. 9A). Tertiary structure modelling clearly shows that PgTCP26-02 contains the bHLH domain (Fig. 9B). To reveal the evolutionary relationship between *TCP* genes in different species, the 9 protein sequences of TCP family members of other species were downloaded from NCBI (Table S7). The phylogenetic tree was constructed using the PgTCP26-02 protein sequence and 9 TCP protein sequences from other species (Fig. 9C). PgTCP26-02 had the closest evolutionary relationship with maize ZmTCP16. At the protein level, PgTCP26-02 and other protein sequences contained the bHLH domain (Fig. 9D).

**Fig. 10:**
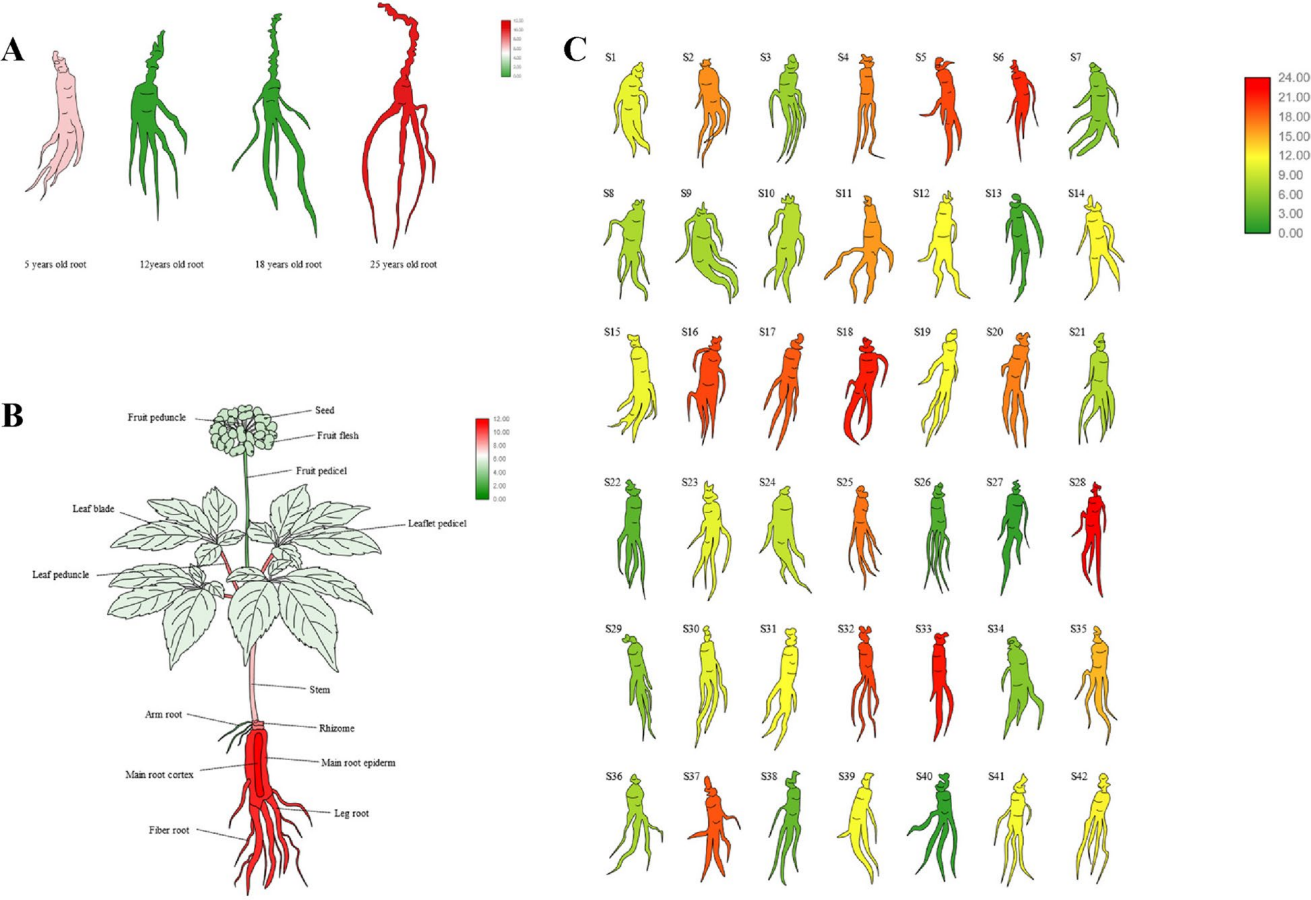
Heatmaps analysis spatiotemporal expression patterns of *PgTCP26-02* gene in *Panax ginseng*. **A** The *PgTCP26-02* gene expressed in the 4 different ages of ginseng roots. Red to green expression decreased in turn. **B** Expression of *PgTCP26-02* gene in 14 different tissues of ginseng. Red to green expression decreased in turn. **C** The *PgTCP26-02* gene expressed in the 42 farm cultivars of 4-year-old ginseng roots. Red, yellow and then green decreased in expression

To study the expression of the *PgTCP26-02* gene in ginseng, we retrieved the expression data of the *PgTCP26-02* gene from roots of 4 different ages of ginseng, 14 different tissues of 4-year-old ginseng and 42 farm cultivars of 4-year-old ginseng. To more intuitively reflect the expression level of the *PgTCP26-02* gene, we drew a gene expression heatmap. Among the 4 different aged stages of ginseng roots, the expression level of the *PgTCP26-02* gene was the highest in 25-year-old ginseng roots and was not expressed in 12-year-old ginseng roots and 18-year-old ginseng roots (Fig. 10A). Among the 14 different tissues of 4-year-old ginseng, *PgTCP26-02* was expressed in all of them, and the expression level of *PgTCP26-02* was higher in the main root epiderm and main root cortex (Fig. 10B). Among the 42 farm cultivars of 4-year-old ginseng roots, the *PgTCP26-02* gene was expressed in all farm cultivars (Fig. 10C).

## Discussion

TCP is a transcription factor family that is widespread and unique to plants and thus has been extensively studied. TCP transcription factors have been shown to be involved in plant growth and development, secondary metabolism and other biological processes in several species, such as *Arabidopsis*, rice, bamboo shoots, tomato, and ginkgo. However, the *TCP* gene family has not been intensively studied in ginseng. In this study, 28 *TCP* gene family members consisting of 78 *TCP* transcription factors named *PgTCP* were identified in the ginseng transcriptome database. Twenty-two *TCP* genes were identified in rice, 24 in *Arabidopsis* [46], 30 in tomato [29], and 12 in ginkgo [24]. Based on evolutionary analysis, *TCP* gene family members in ginseng were also classified into class I (PCF) and class II (CIN and CYC/TB1), and these results suggest that there seems to be no major differences in the number and classification of *TCP* gene family members in plants.

Previous studies have shown that TCP transcription factors almost always have bHLH structures that can bind to DNA and participate in protein interactions and thus in plant growth and development, hormone signalling, and synthesis of secondary metabolites. The protein secondary structure analysis and tertiary structure modelling of *PgTCP* also revealed the existence of a bHLH domain in *PgTCP*, which indicated the functional diversity of the *PgTCP* gene. Ginseng is a tetraploid plant with 24 pairs of chromosomes, and most of the *PgTCP* gene family is distributed on Chinese ginseng chromosomes, with only a few chromosomes that do not have *TCP* transcription factors. Gene replication events are important events that drive the development of new biological functions. However, whole-genome replication of the *PgTCP* gene has also been found in ginseng, so the *PgTCP* gene family has many potential biological functions. Based on the results of GO functional annotation, 77 *PgTCP* transcripts were annotated as molecular functions (MF); thus, these genes may interact with other proteins or regulate the expression of downstream genes by binding enhancers to regulate ginseng growth and development. Seventy-four *PgTCP* transcripts were labelled as biological process (BP), and 47 *PgTCP* transcripts were labelled as cell component (CC), indicating that the *PgTCP* gene is not only a functional but also a structural gene in ginseng. In conclusion, the functions of *PgTCP* transcripts in Jilin ginseng are diverse.

Analysis of the expression pattern of the *PgTCP* gene family yielded several interesting findings. First, the expression levels of *PgTCP* transcripts were analysed in 42 farm cultivars, and most transcripts were expressed very similarly in all cultivars, suggesting broad expression of *PgTCP* transcripts. However, the *PgTCP* gene also has specificity. *PgTCP20-06*, *PgTCP20-03*, and *PgTCP23* were expressed in all farm cultivars, *PgTCP20-3* was strongly expressed in S30, and *PgTCP26-01* and *PgTCP26-02* were highly expressed in the majority of farm cultivars. Second, approximately 62% of *PgTCP* transcripts were expressed at different times in ginseng roots at four different ages (5, 12, 18, and 25 years), and approximately 37% of *PgTCP* transcripts were not expressed at all four ages of ginseng. *PgTCP09-04* had a clear trend of increasing expression over time, and *PgTCP24-21*, *PgTCP24-12*, *PgTCP18* and *PgTCP16-01* showed a clear trend of decreasing expression over time. A total of 33 transcripts (42%), 35 transcripts (44%), 39 transcripts (50%), and 37 transcripts (47%) were expressed in 5-, 12-, 18-, and 25-year-old roots, respectively, and specifically, the *PgTCP* transcript had the lowest expression level in the 5-year-old roots. The specific expression of *PgTCP* transcripts in these 4 different ages indicated that not all *PgTCP* transcripts were structurally expressed in ginseng. Finally, most *PgTCP* genes were found to be expressed with tissue specificity in 14 different tissues of 4-year-old ginseng, and only 14 transcripts (18%) were expressed in all tissues, with *PgTCP20-03* and *PgTCP20-06* being expressed at higher levels in 14 ginseng tissues. In conclusion, the expression of *PgTCP* transcripts in ginseng is spatiotemporally specific.

In 78 transcripts of the *PgTCP* gene family, the expression of most genes is regulated jointly, while only a few genes are regulated independently. Genes of the *PgTCP* gene family are more likely to form a coexpressed interaction network, and some closely related clusters are formed at *P* ≤ 0.05, some of which play a core role in the network, indicating that gene members of the *PgTCP* gene family are still functionally related to each other.

We selected five *PgTCP* genes from each of the three isoforms PCF, CIN and CYC/TB1 as representatives to study their expression under MeJA treatment. The relative expression of genes in class I was downregulated after MeJA treatment. In contrast, the relative expression of most genes in the two isoforms of class II was upregulated after MeJA treatment. This indicates that the gene members of different isoforms of the *PgTCP* gene family differ under abiotic stress treatment. These results not only confirmed that the relative expression level of the *PgTCP* gene after MeJA treatment affected the hairy root of ginseng but also indicated the reliability of the results of systematic analysis in this study. These results also demonstrated that *PgTCP* gene expression in ginseng is not only time-specific but also responsive to the regulation of MeJA. This study provides a theoretical basis for studying *TCP* gene regulation of plant secondary metabolism.

To further investigate the role of *PgTCP* in synthesizing secondary metabolites in ginseng, we identified a gene, *PgTCP26-02,* that is highly related to ginsenoside content in the *PgTCP* gene family. *PgTCP26-02* belongs to the class I (PCF) subfamily, and after MeJA treatment, the expression level of the *PgTCP26-02* gene showed a downwards trend, and the expression level of the *PgTCP26-02* gene in 42 farm cultivars was also negatively correlated with the expression level of key ginsenoside synthesis enzyme-encoding genes. Therefore, we preliminarily determined that the *PgTCP26-02* gene was related to ginsenoside biosynthesis, and the *PgTCP26-02* gene also became the next research object.

Genes control protein synthesis through transcription and translation, and proteins are the embodiment and undertaking of life activities and are closely related to the exercise of biological functions. Therefore, we analysed the protein sequence structure of the *PgTCP26-02* gene, and the tertiary structure of the PgTCP26-02 protein showed that it had a typical bHLH structure. Phylogenetic analysis showed that *PgTCP26-02* had high homology with the *TCP* genes of other species. Through multiple sequence alignment, we found that all *TCP* genes have the bHLH domain, and there are approximately 20 basic regions of amino acid residues in the N-terminus of the bHLH domain and a helix-ring-helix region composed of approximately 40 amino acids in the C-terminus of the bHLH domain. The alkaline region has the ability to bind to the specific DNA sequence E-box (5'-CANNTG-3'), while the two α-helices of HLH can participate in protein‒protein interactions to form homologues or heterodimers. This gives the bHLH transcription factor the dual function of interacting with both DNA and proteins. Therefore, the *PgTCP26-02* gene containing a bHLH domain plays an important regulatory role in the secondary metabolism of ginseng.

Through the analysis of the expression pattern of the *PgTCP26-02* gene, it was found that the *PgTCP26-02* gene had the highest expression in the roots of 25-year-old ginseng, and some studies showed that the saponin content in the roots of older ginseng was also higher than that in the roots of younger ginseng; therefore, we speculated that the *PgTCP26-02* gene was related to the synthesis of ginsenoside. The expression of the *PgTCP26-02* gene is tissue-specific in ginseng, and the expression level of the *PgTCP26-02* gene appears to be the most abundant in ginseng roots. As ginsenoside is the main active ingredient in the root of ginseng [47], our results further support that the *PgTCP26-02* gene might be involved in regulating ginsenoside biosynthesis. We are currently studying the molecular mechanism of *PgTCP26-02* gene involvement in the regulation of ginsenoside synthesis in ginseng, aiming to better understand how *TCP* genes regulate plant secondary metabolism.

## Conclusion

In this study, 28 *PgTCP* genes were screened from *P. ginseng* (ginseng), and their structure, evolution, function, expression pattern, and coexpression network were analysed. Additionally, the response of *PgTCP* genes to MeJA was investigated. Our results suggest that members of the *PgTCP* gene family are functionally diverse, showing differences in expression patterns in terms of tissue and temporal specificity. In addition, the *PgTCP* gene family appears to be involved in the plant response to MeJA treatment, further confirming the role of the *TCP* gene family in the MeJA stress response. The role of the *TCP* gene family in the secondary metabolism of ginseng was further confirmed and provided a theoretical basis for ginseng genetic breeding in the future. Genome-wide identification and integrated analysis of the *TCP* gene family controlling ginsenoside biosynthesis will provide a theoretical basis and enriched genetic resources for in-depth studies on functional genomics in *P. ginseng*.

### Supplementary Information

**Additional file 1: Table S1.** Basic information of *PgTCP* gene family.

**Additional file 2: Table S2.** The identified genes used as evolutionary controls for *PgTPC* gene phylogenetic analysis.

**Additional file 3: Table S3.** The classification, annotation and GO functional categorization of the *PgTCP* gene transcripts.

**Additional file 4: Table S4.** The expressions of the PgTCP gene transcripts in 14 tissues, 42 cultivars' roots and 4 aged roots (TPM).

**Additional file 5: Table S5.** Significance analysis of correlation between *PgTCP* and ginsenoside.

**Additional file 6: Table S6.** Significance analysis of *PgTCPs* expression levels with key enzyme genes.

**Additional file 7: Table S7.** Protein sequences of foreign species.

## Data Availability

All ginseng data used for this study are available at the Sequence Read Archive (SRA) of the National Center for Biotechnology Information (NCBI) under BioProject PRJNA302556. TCP amino acid sequences were downloaded from the Plant Transcription Factor Database (http://planttfdb.gao-lab.org/family.php?fam=TCP). All ginseng materials are available through corresponding authors upon request.
